# A Novel Antimicrobial Coating Represses Biofilm and Virulence-Related Genes in Methicillin-Resistant *Staphylococcus aureus*

**DOI:** 10.3389/fmicb.2018.00221

**Published:** 2018-02-15

**Authors:** Ankita Vaishampayan, Anne de Jong, Darren J. Wight, Jan Kok, Elisabeth Grohmann

**Affiliations:** ^1^Life Sciences and Technology, Beuth University of Applied Sciences Berlin, Berlin, Germany; ^2^Department of Molecular Genetics, University of Groningen, Groningen, Netherlands; ^3^Institute of Virology, Free University of Berlin, Berlin, Germany; ^4^Division of Infectious Diseases, University Medical Center Freiburg, Freiburg, Germany

**Keywords:** antimicrobial surface, MRSA, virulence, biofilm, quorum-sensing, RNA sequencing

## Abstract

Methicillin-resistant *Staphylococcus aureus* (MRSA) has become an important cause of hospital-acquired infections worldwide. It is one of the most threatening pathogens due to its multi-drug resistance and strong biofilm-forming capacity. Thus, there is an urgent need for novel alternative strategies to combat bacterial infections. Recently, we demonstrated that a novel antimicrobial surface coating, AGXX^®^, consisting of micro-galvanic elements of the two noble metals, silver and ruthenium, surface-conditioned with ascorbic acid, efficiently inhibits MRSA growth. In this study, we demonstrated that the antimicrobial coating caused a significant reduction in biofilm formation (46%) of the clinical MRSA isolate, *S. aureus* 04-02981. To understand the molecular mechanism of the antimicrobial coating, we exposed *S. aureus* 04-02981 for different time-periods to the coating and investigated its molecular response via next-generation RNA-sequencing. A conventional antimicrobial silver coating served as a control. RNA-sequencing demonstrated down-regulation of many biofilm-associated genes and of genes related to virulence of *S. aureus*. The antimicrobial substance also down-regulated the two-component quorum-sensing system *agr* suggesting that it might interfere with quorum-sensing while diminishing biofilm formation in *S. aureus* 04-02981.

## Introduction

*Staphylococcus aureus* is an opportunistic pathogen commonly found in the human respiratory tract, nasal areas and skin. It colonizes the anterior nares of approximately 20–25% of the healthy adult population, while 60% are intermittently colonized (Kluytmans et al., 1997; Ellis et al., 2014). Methicillin-resistant *Staphylococcus aureus* (MRSA) is a crucial human pathogen causing infections ranging from skin and soft tissue infections to fatal sepsis (Marathe et al., 2015). It is one of the leading pathogens that cause nosocomial infections (Paniagua-Contreras et al., 2012; Lister and Horswill, 2014); it is resistant to methicillin and many other antibiotics (Marathe et al., 2015), and it is also known to produce thick biofilm (Paniagua-Contreras et al., 2012; Qin et al., 2014). MRSA was shown to cause catheter-associated and other medical devices-related infections (Arciola et al., 2001; Paniagua-Contreras et al., 2012). Eighty percent of prosthetic infections are caused by Staphylococci (Kirmusaoglu, 2016). Its firm attachment to medical devices and host tissues, and its ability to form robust biofilms makes it a cause of chronic infections (Yarwood et al., 2004). *S. aureus* biofilms cause numerous infections in which the accessory gene regulator (*agr*) quorum-sensing system (QS) plays an important role (Yarwood et al., 2004). Around 90% of the infections caused by the bacterium are skin and soft tissue infections, and the *agr*QS system is associated with these infections (Sully et al., 2014).

Multiple drug resistance combined with a thick biofilm makes the treatment and eradication of *S. aureus* infections even more difficult. This entails the urge of development of novel antimicrobials, which could also be potential biofilm inhibitors. Virulence factors of *S. aureus* serve as targets for the newly developed class of biological anti-staphylococcal agents. These targets include, surface bound adhesins, immunoglobulin-binding proteins, surface-associated and secreted proteases, a family of immune-stimulatory exotoxins called ‘superantigens’ (SAgs), and potent leukocidal toxins (Sause et al., 2015).

Metals like copper and silver have been used as antimicrobials since a long time. The use of copper in human civilization is known since the 5th and 6th millennia B.C. Silver was officially approved for use as an antimicrobial agent in the 20th century (Chopra, 2007; Grass et al., 2011; Schäberle and Hack, 2014; Guridi et al., 2015). Copper and copper alloys have also been used as antimicrobials (Warnes and Keevil, 2013). These metals are known to kill bacteria and fungi by a phenomenon called contact killing (Grass et al., 2011) and can be used to coat medical devices as they inhibit biofilm formation of pathogens (Baker et al., 2010). In the 17th century, silver was described as an essential multipurpose medicinal product and the first scientific documentation of its medical use dates from 1901 (Maillard and Hartemann, 2013). However, in 1975, several patients died from a silver resistant *Salmonella* Typhimurium isolate in the Massachusetts General Hospital; this was the first report of silver resistant bacteria (Gupta et al., 1999). Excessive use of silver is questioned due to its toxicity to the environment as well as to the human body (Landsdown, 2010). Silver resistance, like antibiotic resistance in bacteria, prompts us to develop new strategies to control bacterial infections. One such novel, broad-spectrum antimicrobial agent is AGXX^®^.

AGXX^®^ (Largentec GmbH, Berlin, Germany) is a combination of two transition metals, silver and ruthenium which can be galvanically electroplated on various carriers like V2A steel, silver sheets, Polydimethylsiloxane (PDMS), fleece, etc. The coating is conditioned by ascorbic acid and is active against many Gram-positive and Gram-negative bacteria (Guridi et al., 2015). It is not only an efficient antibacterial but also kills yeasts, viruses, and fungi (Landau et al., 2017a,b). The coating was used successfully for the decontamination of industrial cooling and process water (Landau, 2013). As it is only slightly cytotoxic (Bouchard, 2011), it can be incorporated into various medical applications. Although, the exact mode of action of the antimicrobial activity of the coating is not fully understood, it is known that the generation of reactive oxygen species (ROS) plays an important role in making it a potent antimicrobial. The formation of hydrogen peroxide and hydroxyl radicals has been detected by spectroscopic methods (Clauss-Lendzian et al., 2017). Putative formation of other ROS is under investigation. ROS can damage cellular components, including, DNA, lipids and proteins. Superoxide dismutase and catalase are involved in detoxification of ROS (Paraje, 2011).

In this study, we performed total RNA-sequencing of *S. aureus* 04-02981 (MRSA) to investigate differential gene expression after different times of exposure of the pathogen to the antimicrobials AGXX^®^ or Ag. Our data demonstrate that AGXX^®^ likely reduces biofilm formation and virulence in *S. aureus* 04-02981 by interfering with the QS, by down-regulating the expression of toxins like leukocidins (*lukE*) and gamma-hemolysins (*hlgA*), and of genes associated with surface adhesins and capsular polysaccharide.

## Materials and Methods

### Preparation of Antimicrobial Metal Sheets

Silver sheets of 0.125 mm thickness were used as a base material to prepare the antimicrobial metal sheets. Both sides of the silver sheets were etched by immersing them in half-concentrated nitric acid, for 60 s. The silver sheets were cleaned with de-ionized water and galvanically plated with a 0.16 μm ruthenium coating on both sides for 40 s. Then, the sheets were cleaned with de-ionized water, conditioned with ascorbic acid, rinsed with de-ionized water and dried with a paper towel. Prior to use, AGXX^®^, and Ag sheets, used as reference material, were autoclaved at 121°C for 20 min.

### Bacterial Strain and Culture Conditions

*Staphylococcus aureus* 04-02981 (Nuebel et al., 2010) was grown at 37°C in Tryptic Soy Broth [TSB] (Carl Roth GmbH & Co. KG, Karlsruhe, Germany) with constant agitation at 150 rpm or on Tryptic Soy Agar [TSA] (Carl Roth GmbH & Co. KG, Karlsruhe, Germany). Growth inhibition tests on agar surface were performed according to CLSI guidelines for disk diffusion test (Naas et al., 2006). For this assay, 0.25 cm^2^ sheets of Ag and AGXX^®^ were used.

For generation of growth curves, bacteria were pre-cultured overnight, diluted in TSB to an optical density at 600 nm (OD_600_) of 0.05 and incubated for further 8 h either in presence of AGXX^®^ or in the presence of silver (Ag), 24 cm^2^ each in 30 mL medium to obtain a sheet surface to medium volume ratio (A: V) of 0.8. Cultures grown in the absence of a metal sheet served as controls. OD_600_ of the cultures was measured using the Genesys 10S UV-Vis spectrophotometer (Thermo Scientific, China). Colony forming units (CFU) per mL were determined hourly from 0 to 8 h post inoculation. Growth experiments were performed in triplicate with independent biological replicates.

### Biofilm Screening Assay

To study the effect of Ag, and AGXX^®^ on biofilm formation of *S. aureus 0*4-02981, the Crystal Violet Assay was performed without any metal sheet, in presence of Ag (24 cm^2^ uncoated silver sheet) and in presence of AGXX^®^ (24 cm^2^ silver sheet coated with ruthenium for 40 s). The sheet surface: medium volume ratio (A: V) was 0.8 (24 cm^2^ metal sheet: 30 mL medium). The overnight culture of *S. aureus 0*4-02981was diluted to an initial OD_600_ of 0.05. The culture was incubated at 37°C and 150 rpm for 4 h (mid- exponential phase, OD_600_∼1.5). Then, it was transferred to the transparent 96-well plate (Carl Roth GmbH & Co. KG, Karlsruhe, Germany) containing Ag, or AGXX^®^. The plate was incubated at 37°C for 24 h, then the antimicrobial metal sheets were carefully removed and OD_600_ of the cultures was measured. In addition, at this stage, the CFU per mL of the planktonic cultures and the biofilms in presence as well as in absence of the metal sheets were determined. Means of five values each and two biological replicates are given. The biofilm assay was performed according to Schiwon et al. (2013). *Enterococcus faecalis* 12030, a strong biofilm former was used as a positive control (Huebner et al., 1999), and Tryptic Soy Broth (TSB) as a negative control (Schiwon et al., 2013). Biofilm formation was measured in EnSpire Multimode Plate Reader 2300-0000 (PerkinElmer, Turku, Finland) at 570 nm. Normalized biofilm formation was calculated by dividing the biofilm measure at OD_570_ by the bacterial growth at OD_600_. Following criteria were used for the interpretation of the results, ODc = negative control; OD ≤ ODc = non-adherent, ODc ≤ OD ≤ (2 × ODc) = weakly adherent, (2 × ODc) < OD ≤ (4 × ODc) = moderately adherent, (4 × ODc) < OD = strongly adherent, as described in Nyenje et al. (2013). Biofilm inhibitory rates of AGXX^®^ and Ag were calculated using the following equation, as described by Qin et al. (2014).

$$Inhibitory\,\;rate(\%)=\frac{{OD}_{570}(Control)-{OD}_{570}(Sample)*100}{{OD}_{570}\left(Control\right)}$$

Student’s *t*- test was used to check if biofilm inhibition was statistically significant, using SigmaPlot version 11.0 (Systat software, Inc., San Jose, CA, United States^1^) (Wass, 2009).

### Spinning Disk Confocal Microscopy

*Staphylococcus aureus* 04-02981 was grown in TSB overnight at 37°C, 150 rpm, then it was diluted to an OD_600_ of 0.05 and further incubated at 37°C for 4 h (mid-exponential phase, OD_600_ ∼1.5). Then, the culture was transferred to a μ-Dish (μ-Dish 35 mm, low, from ibidi GmbH, Martinsried, Germany) containing Ag, or AGXX^®^ (sheet surface: medium volume ratio = 0.8) and incubated at 37°C for 24 h. The culture was removed from the μ-Dish, and the biofilm on the μ-Dish was washed three times with phosphate buffered saline (PBS). The biofilm was stained for 10 min in the dark with Hoechst 33342 (5 μg/mL) and propidium iodide (1 μg/mL) (Thermo Fisher, Eugene, OR, United States). The staining solution was then replaced with 50% glycerol to prevent movement of bacteria during imaging. Imaging was performed with a Nikon TiE-based Visitron spinning disk confocal microscope using a 100× NA1.45 objective. Fluorescent dyes were excited using 405 nm (Hoechst 33342) and 561 nm (propidium iodide) laser lines and fluorescent emission captured through appropriate filters onto an iXon888 EMCCD detector (Andor, Belfast, United Kingdom). Images were subsequently analyzed using Fiji (ImageJ) version 3.2.0.2.

### Metal Stress and RNA Extraction

Overnight cultures of *S. aureus* 04-02981 were diluted as described above and grown until mid-exponential growth phase (4 h post dilution, OD_600_∼1.5). The cultures were then subjected to metal stress by exposure to AGXX^®^ or Ag sheets (sheet-surface to medium-volume ratio of 0.8) followed by further incubation for 6, 12, 24, 80, and 120 min at 37°C with constant agitation at 150 rpm. As a control, no metal sheet was added to the culture. Cells from 30 mL culture were harvested by centrifugation for 1 min at 10,000 rpm and 4°C in a Heraeus Multifuge X3R Centrifuge (Thermo Electron LED GmbH, Osterode am Harz, Germany). Cell pellets were immediately frozen in liquid nitrogen and stored at -80°C or directly used for RNA extraction using the ZR Fungal/Bacterial RNA MiniPrep^TM^ Kit (ZymoResearch, Freiburg, Germany) following the manufacturer’s instructions. To recover total RNA including small RNAs, 1.5 volumes of absolute ethanol were added in step 5. Finally, total RNA was eluted with 50 μl DNase- and RNase-free water and stored at -80°C. RNA quantity and quality were assessed with a NanoDrop 2000c UV-Vis Spectrophotometer (Thermo Scientific, Osterode am Harz, Germany) as well as on bleach agarose gels. Residual contaminating DNA was eliminated with TURBO DNA-free^TM^ Kit Ambion (Life Technologies, Darmstadt, Germany).

### RNA Sequencing

Total RNA sequencing was done by PrimBio Research Institute, Exton, PA, United States. The protocol was performed in five steps; rRNA removal was done using the Ribo-Zero rRNA Removal Kit (Bacteria) (Illumina, Cat# MRZMB126), followed by library preparation, and templating, enrichment and sequencing.

### RNA-Sequencing Data Analysis

Raw sequencing reads were aligned to the reference genome of *S. aureus* 04-02981, using Bowtie2 (Langmead and Salzberg, 2012) version 2.2.3 with optimal settings for the IonProton^TM^ Sequence. Post-processing of the SAM files into sorted BAM files was carried out with SAMtools (Li et al., 2009, version 1.2-207). The samples AGXX^®^, and Ag were normalized (AGXX^®^-Control, Ag-Control) against the control of the respective time-points. Length normalized confidence interval RPKM (=Reads per Kilobase of transcript per Million mapped reads) values were obtained with Cufflinks (Trapnell et al., 2010). Finally, statistical analysis was carried out using the T-REx RNA-Seq analysis pipeline (de Jong et al., 2015). A gene was considered significantly differentially expressed when the fold change was ≥|2.0| and the false discovery rate (FDR) adjusted *p*-value ≤ 0.05. The data presented in this paper have been deposited at NCBI, and are accessible through GSE103064^2^.

### Reverse Transcription Quantitative PCR (RT-qPCR)

To verify the results obtained from RNA-sequencing, RT-qPCR was performed on five genes detected as highly differentially expressed via RNA-seq. To this end, RNA extracted from *S. aureus* 04-02981 cultures exposed to Ag or AGXX^®^ for 24, and 80 min, was used. First strand cDNA was synthesized with RevertAid^TM^ First Strand cDNA Synthesis kit (Thermo Fisher Scientific Inc., Walham, Germany) as per the manufacturer’s instructions using 120 ng total RNA as template and random hexamer primers. cDNA was diluted with DNase- and RNase-free water and amplified in a LightCycler^®^480 II (Roche Diagnostics GmbH, Mannheim, Germany).

The *agrC*, *lukE*, *sdrC, srrA*, and *cap5A* genes were selected to verify the data obtained through RNA-seq. The gene *gyrB* was used as a control. These genes were amplified using TaqMan chemistry according to the instructions provided in LightCycler^®^ 480 Probes Master Kit (Roche Diagnostics). All RT-qPCR reactions were carried out in a total volume of 20 μL. The amplification step was performed with ‘Quantification’ analysis mode at 95°C for 10 s, with a ramp rate of 4.4°C/s, followed by annealing at the respective annealing temperature for 50 s, with a ramp rate of 2.2°C/s and finally an extension at 72°C for 1 s, with a ramp rate of 4.4°C/s. The amplification step was performed 45 times. All primers and probes used in the study are listed in **Supplementary Table S1**. All RT-qPCR experiments were done in triplicate and each experiment was repeated at least twice. Data were analyzed by LightCycler^®^ 480 Software release 1.5.0 by using the ‘Relative Standard Curve’ method; the standard curves were constructed using genomic DNA from *S. aureus* 04-02981. Data represent expression ratios, calculated by normalizing to the *gyrB* gene and relative to the untreated culture of *S. aureus* 04-02981 which served as the calibrator, as described in ‘Guide to performing Relative Quantitation of Gene expression using real time-quantitative PCR’ by Applied Biosystems. Means of five *C*p values each were used to calculate the relative expression ratio.

### Statistical Analysis

Statistical tests were performed to analyze the significance of the obtained data. Student’s *t*-test was applied to the normalized target, and normalized control values (normalized concentration). The tests were performed and analyzed using SigmaPlot version 11.0 (Systat software, Inc., San Jose, CA, United States^3^) (Wass, 2009).

## Results

### AGXX^®^ Inhibits the Growth of *S. aureus* 04-02981

To analyze the effect of Ag, and AGXX^®^ on the growth of *S. aureus* 04-02981, disk diffusion tests with Ag, and AGXX^®^ were performed in accordance with NCCLS-CLSI guidelines (Naas et al., 2006). The agar plates were monitored at 24 h intervals for 5 days to check if Ag or AGXX^®^ exhibited an inhibitory effect on the pathogen, in the form of a zone of inhibition on the agar plate. The diameter of the inhibition zones was measured in ‘cm.’ The mean diameter of the inhibition zone was calculated to be 1.2 cm for AGXX^®^ while no zone of inhibition was observed for Ag.

To verify the inhibitory effect of AGXX^®^ on *S. aureus* 04-02981 as demonstrated in the agar diffusion tests, experiments in TSB medium were performed measuring the CFU/mL every hour for a period of 8 h, using the A: V ratio (metal mesh: medium volume) of 0.8, as described in Section “Materials and Methods.” As observed in the disk diffusion assay, Ag did not show a significant inhibitory effect on the growth of *S. aureus* 04-02981 in liquid cultures. In contrast, AGXX^®^ had a profound inhibitory effect on this strain. The OD_600_ of *S. aureus* 04-02981 in presence of AGXX^®^ was very low, (OD_600_ AGXX^®^ at t8 = 0.149) as compared to Ag (OD_600_ Ag at t8 = 3.086) and the control (OD_600_ Control at t8 = 3.173) (**Supplementary Table S2**). The CFU/mL of *S. aureus* 04-02981 grown in the batch culture with AGXX^®^ increased from 2.77 × 10^6^ in the 1st hour to 3.99 × 10^10^ in the 4th hour, but then decreased to 1.08 × 10^7^ in the 8th hour. The colony counts of *S. aureus* 04-02981 + AGXX^®^ (after 8 h of growth) were much lower than that of the same strain with Ag (1.27 × 10^11^) or without metal amendment (1.73 × 10^11^) (**Table 1**). These data confirm the antimicrobial effect of AGXX^®^ on *S. aureus* 04-02981.

**Table 1:** Colony forming units (CFU)/mL of *Staphylococcus aureus* 04-02981 (without sheet = control), in the presence of AGXX^®^ or Ag.

Sample	0 h	1 h	2 h
Control	1.27 × 10^6^ ± 2.0 × 10^5^	7.63 × 10^7^ ± 1.5 × 10^6^	8.71 × 10^9^ ± 1.0 × 10^9^
Ag	1.23 × 10^6^ ± 4.7 × 10^5^	6.80 × 10^6^ ± 1.1 × 10^6^	6.23 × 10^9^ ± 1.2 × 10^9^
AGXX^®^	7.67 × 10^5^ ± 4.1 × 10^5^	2.77 × 10^6^ ± 1.4 × 10^6^	4.93 × 10^7^ ± 3.4 × 10^7^
	**3 h**	**4h**	**5 h**
Control	1.11 × 10^10^ ± 1.1 × 10^9^	1.26 × 10^11^ ± 7.5 × 10^9^	**1.73** × **10^11^ ± 3.3** × **10^9^**
Ag	1.02 × 10^10^ ± 1.2 × 10^9^	1.25 × 10^11^ ± 1.0 × 10^10^	**1.27** × **10^11^ ± 6.2** × **10^8^**
AGXX^®^	2.06 × 10^8^ ± 4.7 × 10^7^	3.99 × 10^10^ ± 1.1 × 10^9^	**1.71** × **10^8^ ± 1.6** × **10^7^**
	**6 h**	**7 h**	**8 h**
Control	1.66 × 10^11^ ± 5.7 × 10^9^	1.26 × 10^11^ ± 3.5 × 10^9^	**1.17** × **10^11^ ± 4.4** × **10^9^**
Ag	1.22 × 10^10^ ± 4.6 × 10^8^	1.12 × 10^10^ ± 1.4 × 10^9^	**1.02** × **10^10^ ± 1.4** × **10^9^**
AGXX^®^	1.99 × 10^8^ ± 7.1 × 10^8^	1.34 × 10^7^ ± 1.4 × 10^6^	**1.08** × **10^7^ ± 1.8** × **10^6^**


*The values for 5th hour and 8th hour are bolded because after t = 5h, the CFU values of MRSA + Ag decreased. And until t = 8h, the CFU values for all the three samples (MRSA, MRSA + Ag, and MRSA + AGXX) decreased.*

### AGXX^®^ Strongly Reduces Biofilm Formation of *S. aureus* 04-02981

**FIGURE 1:**
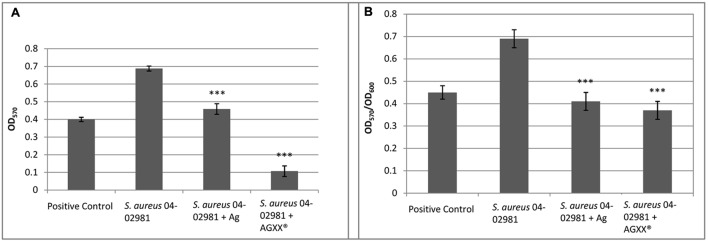
AGXX^®^ reduces biofilm formation of *S. aureus* 04-02981. Mean of five OD values of the biofilm assay with AGXX^®^, Ag, and without metal amendment with standard deviation are shown. **(A)** Shows the biofilm formation by *Staphylococcus aureus aureus* 04-02981, measured at 570 nm; **(B)** shows the biofilm formation (OD_570_) normalized to the bacterial growth (OD_600_) to take the antimicrobial effect of AGXX^®^ into account. Asterisks indicate the *p*-values obtained from *t*-test using SigmaPlot (^∗∗∗∗^*p* < 0.0001, ^∗∗∗^*p* < 0.001, ^∗∗^*p* < 0.01, ^∗^*p* < 0.05) representing the statistical significance of the data.

The effect of AGXX^®^, and Ag on biofilm formation of *S. aureus* 04-02981 was analyzed using the Crystal Violet assay. *E. faecalis* 12030, a strong biofilm former served as a positive control (Huebner et al., 1999), and TSB as the negative control (**Figure 1**). **Figure 1A** shows the biofilm formation by *S. aureus* 04-02981, measured at 570 nm, **Figure 1B** shows the biofilm formation (OD_570_) normalized to the bacterial growth (OD_600_) to take the antimicrobial effect of AGXX^®^ into account.

To determine the bacterial killing activity of AGXX^®^ under these conditions (after 24 h of growth, prior to adding crystal violet), we measured the CFU per mL of the planktonic cultures and the biofilms in the presence as well as in absence of the two different metal sheets.

The following values were obtained for the biofilms: For *S. aureus* 04-02981 without metal sheet (control), 2.34 × 10^9^ ± 8.49 × 10^7^ CFU per mL, for the strain in presence of Ag, 2.13 × 10^9^ ± 2.40 × 10^8^, and in presence of AGXX^®^, 1.80 × 10^4^ ± 1.41 × 10^3^. When we measured the CFU per mL in the respective planktonic cultures, for the control, 2.55 × 10^8^ ± 2.12 × 10^7^, and for the strain in presence of Ag, 2.00 × 10^8^ ± 1.41 × 10^7^ CFU per mL were obtained. However, no colonies were observed in presence of AGXX^®^. Thus, we conclude that in contrast to Ag, all planktonic bacteria were killed by AGXX^®^ and after exposure to AGXX^®^, only a drastically reduced number of bacteria (1.80 × 10^4^ CFU per mL) survived in the biofilm in comparison to Ag (2.13 × 10^9^ CFU per mL).

In summary, the biofilm formation measures normalized to the bacterial growth show that AGXX^®^ reduced biofilm formation of *S. aureus* 04-02981 by 46%, whereas the inhibitory effect of Ag on biofilm formation was less pronounced (41%).

**FIGURE 2:**
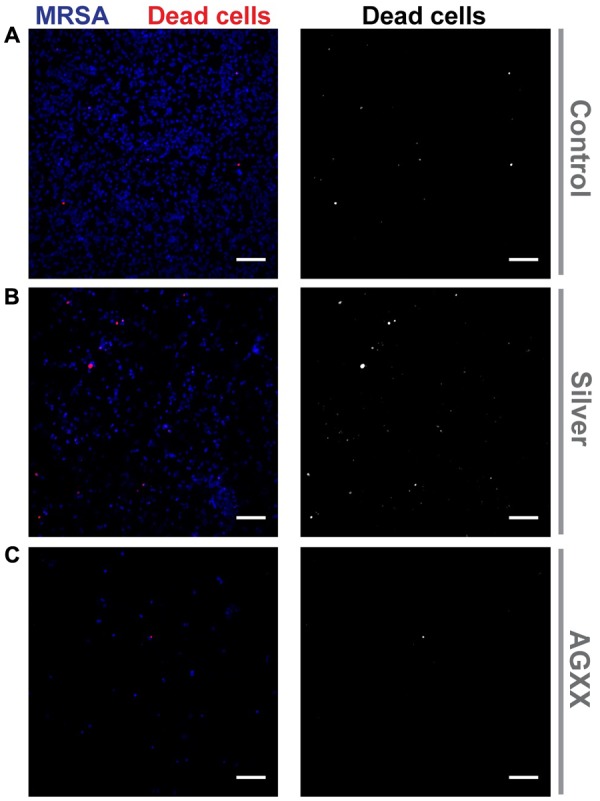
Confocal images of biofilm formation by *S. aureus* 04-02981 (MRSA). The pathogen was grown on sterile cover slips for 24 h with the following conditions: **(A)** without antimicrobial sheet, **(B)** with a silver sheet or **(C)** with an AGXX^®^ sheet. Biofilms were then stained with Hoechst 33342 (to mark out *S. aureus* 04-02981; blue) and propidium iodide (to identify dead cells; red) followed by acquisition of small *Z*-stacks (500 nm spacing) through the biofilms using a confocal microscope. Images show an average of Z-projection (average of 4–5 Z planes containing the biofilm) of the fluorescence signal through the biofilms with the propidium iodide staining shown alone in the images on the right (gray scale images). Scale bars = 10 μm.

The strong reduction of biofilm formation by AGXX^®^ was confirmed by Hoechst 33342/propidium iodide staining of biofilms grown for 24 h in presence of AGXX^®^, Ag and without antimicrobial sheet (**Figure 2**). The inhibitory effect of Ag was also clearly visible, although it was less distinct.

### AGXX^®^ Strongly Induces Stress Response and Represses Pathogenesis in *S. aureus* 04-02981

**FIGURE 3:**
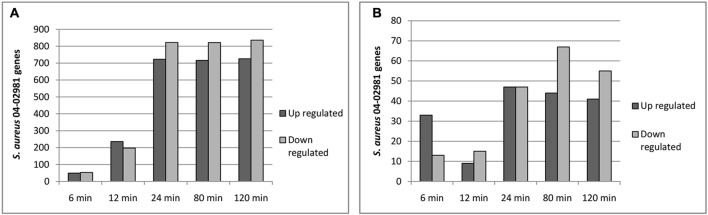
**(A,B)** Show the number of differentially expressed genes in *S. aureus* 04-02981 on exposure to AGXX^®^ **(A)** and Ag **(B)** for the indicated time-period, compared to control (*S. aureus* 04-02981 grown without a metal sheet).

The raw RNA sequence data obtained were aligned to the *S. aureus* 04-02981 genome. High sequencing depth was achieved as a mean value of ∼12.4 million reads was obtained. The numbers of reads per sample ranged from ∼8.4 million reads (Ag_24) to 175 million reads (Control_120) (**Supplementary Table S3** and **Supplementary Figure S1**). From the data, it is clear that the antimicrobial coating has a strong impact on the transcriptome of *S. aureus* 04-02981. In total, 2864 genes were differentially expressed in *S. aureus* 04-02981 on exposure to AGXX^®^ and Ag (**Supplementary Table S4**). The number of differentially expressed genes in presence of AGXX^®^ or Ag at different time-points is presented in **Figure 3**.

From **Figure 3A**, it can be seen that the number of differentially expressed genes at t24, t80, and t120 was quite similar. The maximum impact of AGXX^®^ on the transcriptome of *S. aureus* 04-02981 was reached already after exposure for 24 min (723 genes up-regulated and 823 genes down-regulated) and remained nearly the same after exposure for 80 min (716 genes up- and 822 genes down-regulated), and 120 min (726 genes up- and 836 genes down-regulated). The lowest number of genes was differentially expressed at t6.

**FIGURE 4:**
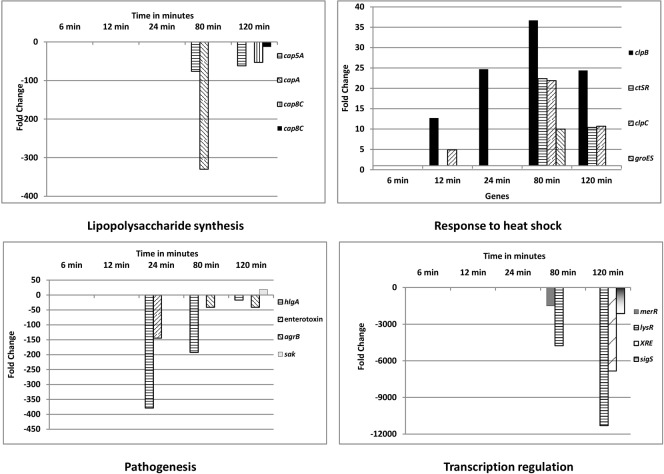
Gene Ontology (GO) categories of differentially expressed genes of *S. aureus* 04-02981 exposed to AGXX^®^ for different time-periods. Only significantly differentially expressed genes, which are likely relevant in the response of *S. aureus* to AGXX^®^, are presented. Details on the other genes are provided in the **Supplementary Table S5**, along with all of the other GOs, Gene IDs, and fold-change in gene expression, for each time-point. Capsular polysaccharide synthesis mediating *cap* genes are shown in ‘Lipopolysaccharide synthesis.’ Genes encoding chaperone ClpB (*clpB*), transcriptional regulator CtsR (*ctsR*), ATP-dependent Clp protease ATP-binding subunit ClpC (*clpC*), and chaperonin GroES (*groES*) are presented in the panel showing response to heat shock. In the pathogenesis panel, the genes for γ-hemolysin subunit A (*hlgA*), enterotoxin, accessory gene regulator subunit B (*agrB*), and staphylokinase (*sak*) are presented. Among the transcriptional regulator genes, that of the LysR family transcriptional regulator (*lysR*), MerR family transcriptional regulator (*merR*), XRE family transcriptional regulator (*XRE*) and RNA polymerase sigma factor SigS (*sigS*) are shown.

The differentially expressed genes were categorized as per Gene Ontology (GO) using the GSEA_Pro option in the RNA-Seq analysis section in the T-REx RNA-Seq analysis pipeline (de Jong et al., 2015). Several GOs were obtained via GSEA_Pro, namely, oxidoreductase process, lipopolysaccharide synthesis, ATP binding, membrane transport, metabolism, metal binding, pathogenesis, transcription regulation, response to heat shock, iron-siderophore transporter activity, serine protease activity, etc. (**Supplementary Table S5**). In the GO “lipopolysaccharide synthesis,” the *cap* genes mediating capsular polysaccharide synthesis (*cap*5A, *cap*A, and *cap*8C) were all down- regulated. Genes (*clp*B, *cts*R, *clp*C, and *groE*S) involved in response to heat shock were up-regulated. Among the genes related to virulence (pathogenesis), 10 out of 11 genes were down-regulated, while only one gene was up-regulated at t120 (staphylokinase, a plasminogen activator). Among the responding transcriptional regulator genes, nine were up-regulated and 25 were down-regulated. **Figure 4** shows the differential expression of these GOs in *S. aureus* 04-02981 exposed to AGXX^®^.

*hlgA* (SA2981_RS09385) was the most differentially expressed gene associated with virulence, it was down-regulated at t24 (378 fold), at t80 (192 fold), and at t120 (16 fold). The protein encoded by *hlgA* functions as a two-component toxin along with leukocidins in the lysis of erythrocytes (Gouaux et al., 1997). Among the transcriptional regulators, the gene of the LysR family transcriptional regulator, *lysR* was the most significantly influenced one by AGXX^®^, being down-regulated about 4700 fold at t80, and about 11,000 fold at t120. One of the LysR family transcriptional regulators, HutR is involved in metabolic processes of *S. aureus* (Ibarra et al., 2013). AGXX^®^ had the highest impact on the expression of *cap*A, of all the genes mediating capsular polysaccharide synthesis. *cap*A was down-regulated by 329 fold at t80. Among the most differentially expressed genes in response to heat shock was *clp*B. It is a member of the stress-induced multi-chaperone system and works with DnaK, DnaJ, and GrpE in the recovery of the cell from heat-shock damage (Frees et al., 2005). Among the genes in the GO families influenced by AGXX^®^, only those involved in enterotoxin (SA2981_RS09440), and staphylokinase production were also influenced by Ag, by -533 fold, and -2 fold, respectively, at t80 (**Supplementary Table S6**). In addition to the GO families, the effect of AGXX^®^, and Ag on the expression of operons in the pathogen was analyzed using the GSEA_Pro option on the T-REx pipeline. The results are presented in **Supplementary Tables S7**, **S8**, respectively.

### AGXX^®^ Represses the Expression of Biofilm and Virulence-Associated Genes

We checked the effect of AGXX^®^, and Ag on the expression of genes associated with biofilm formation and virulence in *S. aureus* 04-02981. Many genes that are known to be crucial for biofilm formation and virulence were differentially expressed on exposure to AGXX^®^ while Ag had an effect on just a few of them. The genes affected by AGXX^®^ encode virulence factors, methicillin resistance, surface adhesins, capsular polysaccharide, two-component systems, and other biofilm- associated genes, as well as toxins (**Table 2**).

**Table 2:** Differential expression of biofilm, and virulence-associated genes in *S. aureus* 04-02981 on exposure to AGXX^®^.

Locus tag	Abbreviation	Description	6 min	12 min	24 min	80 min	120 min
SA2981_RS10640	*agrD*	Accessory gene regulator D			-8.9	-11.9	-17.5
SA2981_RS10645	*^∗^agrC*	Histidine kinase of the competence regulon ComD			-9.9	-7.7	-7
SA2981_RS10635	*agrB*	Accessory gene regulator B		-5.7	-18.3	-40.7	-40.8
SA2981_RS10650	*agrA*	Two-component system, LytR family, response regulator AgrA			-2.2		
SA2981_RS05970	PSM- β	Phenol-soluble modulin Beta				-10.5	-22.7
SA2981_RS05965	PSM- β	Phenol-soluble modulin Beta				-10.2	-12.4
SA2981_RS10825	*sigB*	RNA polymerase Sigma-B factor			-2.3	-4	-4.9
SA2981_RS07680	*^∗^srrA*	DNA-binding response regulator SrrA		-4.6	-9.1	-4.6	-5.3
SA2981_RS00190	*mecA*	mecA-Penicillin- binding Protein 2		-2.3	-5.5		
SA2981_RS12040	*sarR*	Transcriptional regulator SarR					
SA2981_RS06390	*codY*	GTP-sensing transcriptional pleiotropic repressor CodY			-2.3	2.4	3.2
SA2981_RS00550	*sarH1*	Staphylococcal accessory regulator A			-2.4		
SA2981_RS05325	*FmtA*	FmtA protein involved in methicillin resistance				-2.6	
SA2981_RS05940	*arcD*	Arginine/ornithine antiporter ArcD					2.4
SA2981_RS00770	*capF*	Capsular polysaccharide synthesis enzyme Cap8F			-4.8	-3.1	-2
SA2981_RS05275	*sspb*	Staphopain B precursor				-2.3	
SA2981_RS13390	*cidA*	Holin-like protein				2.8	8.2
SA2981_RS13925	*arcA*	Arginine deiminase	-2.6	-11.8	-114.7	-20	-4.6
SA2981_RS03620	*saeR*	two-component system, OmpR family, response regulator SaeR					2.3
SA2981_RS06960	*FmtC*	Protein involved in methicillin resistance/L-lysine modification of phosphatidylglycerol			2.3		
SA2981_RS05900	hemolysin II	Alpha-hemolysin precursor				3.8	3.7
SA2981_RS01335	*lrgA*	Antiholin-like protein		-2.6	-3.1		-3.7
SA2981_RS00745	*^∗^capA*	Capsular polysaccharide synthesis enzyme Cap5A			-34.9	-76.9	-61.9
SA2981_RS00750	*capB*	Tyrosine-protein kinase EpsD/capsular polysaccharide synthesis enzyme		2.4	-4.9	-29	-38.1
SA2981_RS00755	*capC*	protein-tyrosine phosphatase/capsular polysaccharide synthesis enzyme		2.8	-15	-20205	-52.9
SA2981_RS13940	*aur*	Zinc metalloproteinase precursor/aureolysin			-12.4		
SA2981_RS02875	*^∗^sdrC*	Serine-aspartate repeat-containing protein C			-13.4		-9.8
SA2981_RS02035	Exotoxin 6	Superantigen-like protein			-8.7	4	-12.6
SA2981_RS13920	*arcB*	Ornithine carbamoyltransferase			-16.3	-26.1	-8.6
SA2981_RS05715	*isdC*	NPQTN cell wall anchored protein IsdC				-5	-9.1
SA2981_RS05735	*srtB*	Sortase B				-8.3	-22.4
SA2981_RS14090	*icaD*	Polysaccharide intercellular adhesin (PIA) biosynthesis protein		54.7			5.6
SA2981_RS14085	*icaA*	Polysaccharide intercellular adhesin (PIA) biosynthesis *N*-glycosyltransferase			35.7	29.9	26.5
SA2981_RS14095	*icaB*	Polysaccharide intercellular adhesin (PIA) biosynthesis deacetylase				-102.4	7.8
SA2981_RS09385	*^∗^lukE*	Leukotoxin/leukocidin			-378.9	-192.2	


**^∗^Genes selected for validation via RT-qPCR*.*

**FIGURE 5:**
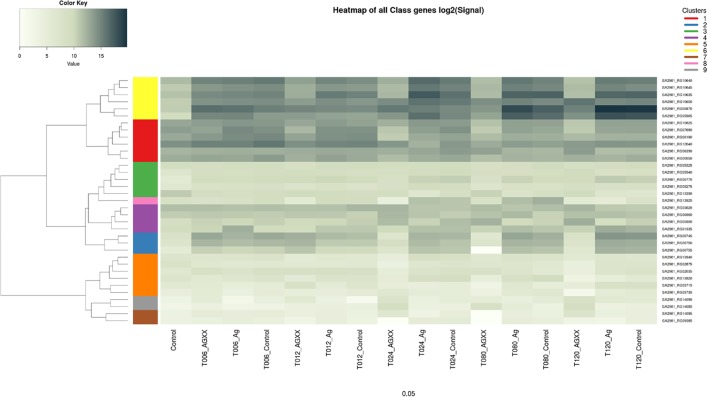
Heatmap of differential expression of biofilm, and virulence-associated genes in *S. aureus* 04-02981. The genes are clustered as indicated by the dendrograms on the left side of the heatmap. Yellow represents genes *agrD, ^∗^agrC, agrB, agrA*, and *PSM*-β, red represents genes *sigB, ^∗^srrA, mecA, sarR, codY*, and *sarH1*. Green color is for genes *fmtA, arcD, capF*, *sspb, cidA*, pink represents *arcA* while purple is for *saeR, fmtC*, hemolysin II, and *lrgA* genes. Blue represents genes mediating capsular polysaccharide synthesis, namely, ^∗^*capA*, *capB, capC. aur, ^∗^sdrC*, exotoxin 6, *arcB, isdC*, and *srtB* are shown in orange. Gray represents *icaD*, and *icaA* and brown color represents *icaB*, and ^∗^*lukE* genes. ^∗^Indicates genes selected for RT-qPCR.

Upon exposure to AGXX^®^, the QS system genes *agrA*, *agrB*, *agrC*, and *agrD* of *S. aureus* 04-02981 were all down-regulated. Genes involved in the synthesis of capsular polysaccharide were also down-regulated. In general, the response of *S. aureus* 04-02981 to AGXX^®^ was clearly visible after 24 min of exposure time. Genes encoding adhesins, *isdC, srtB*, and *sdrC* were also down-regulated. The *mecA* gene was down-regulated at t24. The up-regulation of genes inducing biofilm formation in *S. aureus*, such as *saeR* (2.3 fold at t120), *icaA* (36 fold at t24, 29 fold at t80 and 27 fold at t120), *icaB* (8 fold at t120) and *icaD* (55 fold at t12, and 6 fold at t120) was intriguing. The genes *icaB*, *icaA*, and *icaD* are involved in *ica*-dependent biofilm formation. In addition, other key genes associated with biofilm formation and virulence, such as, *codY*, *srrA*, *luxS*, and genes for toxins like leukocidins, enterotoxins, hemolysins, were all differentially expressed at least at one of the time-points (**Figure 5**). Description of all locus tags and Gene IDs shown to the right of the heatmap is given in **Table 2**.

In general, it was observed that AGXX^®^ had a huge impact on the transcriptome of *S. aureus* 04-02981, in particular at the later time-points 24, 80, and 120 min. In contrast, the effect of Ag was much less pronounced as already visible in the growth kinetics and to a lesser extent in the biofilm assays. Although, quite a number of *S. aureus* 04-02981 genes were differentially expressed upon exposure to Ag, only very few belong to the group of biofilm or virulence-associated genes. Among those, which were significantly differentially expressed in the presence of Ag, were *fmtC*, which is associated with methicillin resistance (approximately 3 fold up-regulated at t80; in the presence of AGXX^®^ it was 2 fold up-regulated at t24), transcriptional regulator *sarR* (approximately 3 fold down-regulated at t24; not differentially expressed in the presence of AGXX^®^), the gene of the holin-like protein CidA (approximately 4 fold down-regulated at t24; ∼2 and ∼8 fold up-regulated at t80 and t120, respectively, with AGXX^®^), the arginine deaminase gene *arcA* (approximately 6 fold down-regulated at t120 and 4.6 fold down-regulated with AGXX^®^), the hemolysin II gene (approximately 2 fold down-regulated at t24 and approximately 3 fold down-regulated at t120; ∼3.7 fold up-regulated with AGXX^®^ at t80 and t120) and the gene of the antiholin-like protein lrgA (approximately 6 fold up-regulated at t6 with Ag, in the presence of AGXX^®^, it was ∼3- to 3.7 fold down-regulated at t24, t80, and t120).

### Validation of RNA-Sequencing Data Using RT-qPCR

**FIGURE 6:**
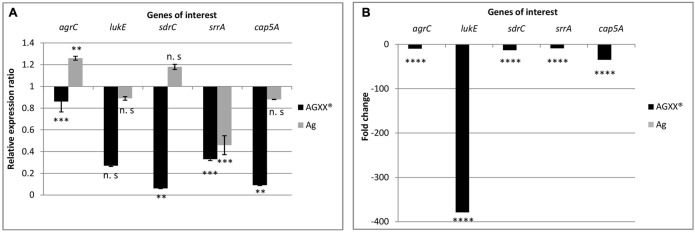
Differential expression of *agrC*, *lukE*, *sdrC*, *srrA*, and *cap5A* in *S. aureus* 04-02981 on 24-min exposure to Ag or AGXX^®^, obtained via RT-qPCR **(A)**. Expression ratio of the genes of interest in *S. aureus* 04-02981 on exposure relative to control (untreated culture of *S. aureus* 04-02981) normalized to *gyrB*. **(B)** Shows differential expression of *agrC*, *lukE*, *sdrC*, *srrA*, and *cap5A* in *S. aureus* 04-02981 on 24-min exposure to Ag or AGXX^®^, obtained via RNA-seq as fold change. Error bars indicate standard deviation. Asterisks indicate *p*-values showing statistical significance. They were obtained from *t*-test using SigmaPlot 11.0 (^∗∗∗∗^*p* < 0.0001, ^∗∗∗^*p* < 0.001, ^∗∗^*p* < 0.01, ^∗^*p* < 0.05; n.s, not significant).

**FIGURE 7:**
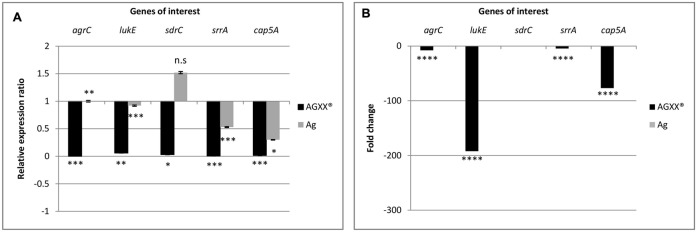
Differential expression of *agrC*, *lukE*, *sdrC*, *srrA*, and *cap5A* in *S. aureus* 04-02981 on 80-min exposure to Ag or AGXX^®^, obtained via RT-qPCR. Expression ratio of the genes of interest in *S. aureus* 04-02981 on exposure relative to control (untreated culture of *S. aureus* 04-02981) normalized to *gyrB* are shown **(A)**. **(B)** Shows differential expression of *agrC*, *lukE*, *sdrC*, *srrA*, and *cap5A* in *S. aureus* 04-02981 on 80-min exposure to Ag or AGXX^®^, obtained via RNA-seq as fold change. Error bars indicate standard deviation. Asterisks indicate *p*-values showing statistical significance. They were obtained from *t*-test using SigmaPlot 11.0 (^∗∗∗∗^*p* < 0.0001, ^∗∗∗^*p* < 0.001, ^∗∗^*p* < 0.01, ^∗^*p* < 0.05; n.s, not significant).

From the RNA-seq data, we observed that AGXX^®^ affected genes encoding two-component systems, surface adhesins, capsular polysaccharides, and toxins. In total, five, highly differentially expressed genes encoding these functions were selected to validate the RNA-seq derived transcriptional response of *S. aureus* 04-02981 to exposure to Ag or AGXX^®^. The validation experiment was performed on RNA extracted from *S. aureus* 04-02981 cultures exposed for 24, and 80 min to Ag or AGXX^®^ since the selected genes were most differentially expressed at these time-points. The five selected genes were, *agrC*, and *srrA* which are part of the two-component systems AgrCA and SrrAB, respectively (Baker et al., 2010; Wu et al., 2015), *lukE* which encodes a toxin (Liu et al., 2016), *sdrC* specifying a surface adhesin (Barbu et al., 2014), and *cap5A* mediating the synthesis of capsular polysaccharides (Qin et al., 2014). *gyrB* was used as the house-keeping gene (Smith et al., 2010; Cheung et al., 2011). **Figures 6**, **7** show the results of these experiments.

After exposure to AGXX^®^ for 24 min, all five genes were down-regulated both in RNA-seq analysis and in RT-qPCR studies as can be seen in **Table 2**, and **Figure 6**. However, after exposure to AGXX^®^ for 80 min, *sdrC* was down-regulated in RT-qPCR assays but it was not differentially expressed in RNA-seq. All the other genes were down-regulated in both approaches as seen in **Table 2** and **Figure 7**, respectively. On exposure to AGXX^®^ for 24 min, *sdrC* was the most down-regulated gene followed by *cap5A*, *lukE*, *srrA*, and *agrC*, whereas after 80 min, *agrC* was the most down-regulated gene followed by *srrA, lukE, cap5A*, and *sdrC*. On exposure to Ag for 24 min, *srrA* was the most down-regulated gene, whereas *agrC* was the most up-regulated gene, and after 80 min, *cap5A* was the most down-regulated gene while *sdrC* was the only up-regulated gene, as observed in the RT-qPCR experiments.

## Discussion

Multiple drug resistant, biofilm forming nosocomial pathogens such as MRSA pose a severe threat to public health demanding the development of novel antimicrobials as well as potent biofilm inhibitors. AGXX^®^ is an effective antimicrobial that is active against many Gram-positive and Gram-negative bacteria (Guridi et al., 2015). AGXX^®^ has been demonstrated to kill *S. aureus* 04-02981 as shown here by disk diffusion assay and growth kinetics experiments. In addition, AGXX^®^ inhibited biofilm formation of *S. aureus* 04-02981 by ∼46%. Moreover, for all time-points examined, the number of differentially expressed *S. aureus* 04-02981 genes was much higher upon exposure to AGXX^®^ (in total 2391) than to Ag (317). For t120, the time-point showing the highest number of differentially expressed *S. aureus* 04-02981 genes, 1562 genes were differentially expressed in presence of AGXX^®^, while only 96 genes were affected by Ag.

Up-regulation of genes of Gene Ontology (GOs) groups “response to heat shock” and “oxidoreductases” involved in oxidative stress response, and down-regulation of genes of GOs “pathogenesis” and “lipopolysaccharide synthesis” involving genes mediating capsular polysaccharide synthesis important for biofilm formation, point to a role of AGXX^®^ as an antimicrobial and potent biofilm inhibitor. Together with results of a recent study where we have shown that the QS system of *S. aureus* 04-02981, *agr* was completely repressed after 4 h of exposure to AGXX^®^ (Probst et al., 2016), we propose that AGXX^®^ acts as a potential biofilm inhibitor. In *S. aureus*, two main mechanisms of biofilm formation are known, namely *ica*-dependent biofilm formation, which involves the production of polysaccharide intercellular adhesin (PIA), and *ica*-independent biofilm formation (Kirmusaoglu, 2016). Here we show that, in the presence of AGXX^®^, *icaA, icaD* were up-regulated and *icaB* was down-regulated. *icaA* and *icaD* contribute to the production of PIA (polymer). *icaD* transfers PIA to the cell surface of the bacteria while *icaB* deacylates PIA by fixing PIA to the outer surface of the bacteria (Kirmusaoglu, 2016). In our study, intercellular adhesion biosynthesis *N*-deacetylase, *icaB* gene was down-regulated at t80 by ∼100 fold. The structural development of exopolysaccharide-based biofilm requires deacetylation of PIA (Arciola et al., 2015). Since *icaB* was strongly down-regulated at t80, deacetylation of PIA probably does not occur which would obstruct the development of an exopolysaccharide-based biofilm. Fitzpatrick et al. (2005) showed that biofilm formation was unaffected in an *icaADBC* operon-deleted MRSA strain, while the same mutation in a methicillin sensitive strain of *S. aureus* (MSSA) impaired biofilm formation, suggesting strain-specificity in *ica*-dependent biofilm formation.

A two-component system associated with *ica*-dependent biofilm formation is SrrAB that acts as an autoregulator of biofilm formation. Deletion of *srrAB* inhibited *S. aureus* biofilm formation under oxic as well as microaerobic conditions (Wu et al., 2015). In our study, *srrA* was down-regulated 4 to 5 fold after 24, 80, and 120 min of exposure to AGXX^®^.

Global regulatory systems such as the *agr QS* system are among the best-studied factors involved in *ica*-(PIA) independent biofilm formation. Other proteins involved in such biofilms are SasG, SasC, Protein A, FnbB, FnbA, ATLA or ATLE, SdrG, SdrC, SdrD, biofilm associated protein (Bap) and lipoteichoic acid (Kirmusaoglu, 2016). We observed that two of these genes were down-regulated when AGXX^®^ was present, namely *sdrD* and *sdrC*, *sdrC* was down-regulated 13- to 10 fold at t24 and t120, while *sdrD* was down-regulated 2 to 3 fold at t24 and t80. Moreover, the expression of lipoteichoic acid synthase, an enzyme responsible for the synthesis of lipoteichoic acid (Karatsa-Dodgson et al., 2010) was down-regulated approximately 4 fold after 24, 80, or 120 min of AGXX^®^ presence. These data suggest that AGXX^®^ might be working in an *ica*-independent manner to inhibit biofilm formation.

The *agr* locus contains five genes, *agrA*, *agrB*, *agrC*, *agrD*, and *hld*. On exposing *S. aureus* 04-02981 to AGXX^®^, only *hld* was not differentially expressed at any time-point, while all the other four genes were significantly down-regulated. The *agr* gene cluster regulates the expression of virulence factors such as phenol soluble modulins (PSMs), proteins that are closely associated with human skin and soft tissue infections (SSTIs) (Sully et al., 2014). “AgrD is a precursor peptide of autoinducer peptide (AIP)” (Quave and Horswill, 2014), AgrB is a membrane protease, which is involved in proteolytic processing and export of AgrD. It is also involved in AIP production (Njoroge and Sperandio, 2009; Quave and Horswill, 2014). AgrBD produce and secrete AIPs. AgrC, a sensor histidine kinase is activated when AIPs bind to AgrC. As a consequence, AgrC undergoes phosphorylation to activate AgrA, which is a DNA-binding response regulator (Njoroge and Sperandio, 2009). In our study, the *agrB* gene was the most down-regulated, at t80, and t120 (approximately 41 fold in both cases), while *agrA* was differentially expressed only at t24 (2 fold down-regulated). At t12, only *agrB* was differentially expressed, approximately 6 fold down-regulated. None of the *agr* genes was differentially expressed at t6. PSMs are staphylococcal toxins playing a role in acute infection (Kirmusaoglu, 2016); they are required for maturation and detachment of biofilm (Ma et al., 2012). PSMs were also down-regulated in presence of AGXX^®^ by ∼10 fold at t80, and by 12 and 23 fold at t120. *agr* also regulates the expression of *sspB* which encodes a cysteine protease. *sspB* is positively associated with biofilm formation (Ma et al., 2012). It was down-regulated by 2.3 fold at t80. Inactivation of the alternative sigma factor SigB decreases biofilm formation in *S. aureus* (Ma et al., 2012). In presence of AGXX^®^, *sigB* was down-regulated 2–5 fold at the longer exposure times (t24, t80, and t120). In summary, down-regulation of all of the genes mentioned in this paragraph will likely reduce biofilm formation by *S. aureus*.

The two component systems, AgrCA and SaeRS influence biofilm formation in *S. aureus*, by the production of PSMs and by suppressing the synthesis of extracellular proteases, respectively (Baldry et al., 2016). The extracellular proteases degrade proteins that are important for biofilm formation (Baldry et al., 2016). In *S. aureus*, the *saeRS* system regulates the production of many virulence factors such as leukocidins, superantigens, proteases, surface proteins, and hemolysins (Liu et al., 2016). The gene for LukE, which enables *S. aureus* evasion from phagocytic cells by damaging the phagocytes was strongly down-regulated at t24 (379 fold) and t80 (192 fold). SplA is a serine protease, which is directly controlled by the *saeRS* system. *splA* was down-regulated 135 fold after 80 min of AGXX^®^ presence. Mutations in genes for extracellular proteases (*spl*ABCDEF) in *S. aureus* SH1000 induced an increase in extracellular protease activity, which was associated with a reduction in biofilm formation (Chen et al., 2013). These facts taken together with *saeRS* not being differentially expressed at any time-point in the presence of AGXX^®^, except for a slight 2.3 fold up-regulation of *saeR* at t120, might suggest that *saeR* is not expressed in the mid exponential phase of growth of *S. aureus* 04-02981.

Capsular polysaccharides are also possible targets of the *saeRS* system (Liu et al., 2016). They play an important role in the virulence of the organism (Tuchscherr et al., 2010). The synthesis of capsular polysaccharides is mediated by the *cap5ABCFG* genes (Qin et al., 2014). Among these genes, only *cap*G was not differentially expressed, all other genes were significantly down-regulated, especially at t24, t80, and t120, suggesting a role of AGXX^®^ in repression of virulence in *S. aureus* 04-02981.

Another QS system, which significantly influences biofilm formation and virulence in Staphylococci is the *luxS* system. *lux*S impacts biofilm formation in a similar way as *agr* does, but by regulating different factors. *lux*S negatively regulates biofilm formation via cell-cell interactions based on autoinducer 2 secretion (Xu et al., 2006). The gene was 2.9 fold up-regulated at t24 in the presence of AGXX^®^.

In addition, the genes *isdC*, *srtB*, *sdrC*, encoding adhesins, were all down-regulated in the pathogen exposed to AGXX^®^. Iron regulated surface determinant IsdC is necessary for the primary attachment of *S. aureus* to surfaces such as polystyrene, as well as for the accumulation phase of biofilm formation; as such, it induces biofilm formation (Missineo et al., 2014). IsdC is anchored to the cell wall by sortase B (Hammer and Skaar, 2011). Serine-aspartate repeat containing protein C precursor (SdrC) assists bacteria in adhering to surfaces and promotes biofilm formation (Barbu et al., 2014). In *S. aureus* 04-02981 exposed to AGXX^®^, *isdC* was down-regulated by 5 and 9 fold at t80 and t120, respectively. The sortase B gene *srtB* was also down-regulated in cells treated with AGXX^®^, at t80 (8 fold) and t120 (22 fold). *sdrC*, too, was down-regulated some 10 to 13 fold at t24 and t120. Thus, we suggest that AGXX^®^ inhibits biofilm formation in *S. aureus* 04-02981, also by repressing the expression of adhesins.

Reverse transcription quantitative PCR assays were performed on RNA extracted from *S. aureus* 04-02981 cultures exposed to Ag or AGXX^®^ for 24 min, and 80 min to validate the RNA-seq data. In RT-qPCR, on exposure to AGXX^®^ for 24 min, *agrC*, *sdrC*, *srrA*, and *cap5A* were statistically significantly down-regulated, whereas the down-regulation of *lukE* was not statistically significant. In agreement with these data, the five genes were also significantly down-regulated in RNA-seq. By contrast, none of the five genes was significantly differentially expressed after 24 min in presence of Ag, as determined by RNA-seq, whereas RT-qPCR revealed a statistically significant down-regulation of *srrA* and a statistically significant up-regulation of *agrC*. The difference in expression of the other three genes *lukE*, *sdrC*, and *cap5A* was statistically not significant. When *S. aureus* 04-02981 was exposed to AGXX^®^ for 80 min, all the five genes were down-regulated in RT-qPCR. The effect was statistically significant while in RNA-seq all genes were significantly down-regulated except *sdrC*. On exposure to Ag for 80 min, only *sdrC* was non-statistically significantly up-regulated. Thus, the trends in gene expression of *S. aureus* 04-02981 on exposure to AGXX^®^ observed in RNA-seq and in RT-qPCR were similar.

In previous studies by others, differential gene expression of *S. aureus* in planktonic and biofilm mode has been examined. Resch et al. (2005) observed that in biofilms, genes encoding polysaccharide intercellular adhesin, and enzymes associated with cell envelope synthesis were significantly up-regulated (Resch et al., 2005). To combat biofilms, many metals have been tested for their capacity to inhibit bacterial biofilm formation. Specifically, silver nanoparticles have received much attention with respect to their antimicrobial nature. However, the minimum concentration of silver nanoparticles (AgNPs) required to eliminate biofilm formation is considered to have toxic effects on mammalian cells (Loo et al., 2016). They studied the effect of AgNPs and curcumin nanoparticles (Cur-NPs) on *S. aureus* and discovered that the combination of both nanoparticles was more effective than the individual AgNPs or Cur-NPs. Curcumin interferes with the QS system as was observed by the down-regulation of genes involved in QS, upon exposure to the substance (Loo et al., 2016). Ma et al. (2012) investigated the effect of two novel anti-virulence compounds on growth and biofilm formation of *S. aureus*. The compounds inhibited biofilm formation by repressing genes associated with biofilm formation such as *lrgA, sdrD, sspB, sigB, codY*, which were also down-regulated in our studies at least at one of the five time-points (Ma et al., 2012).

In summary, based on our findings, we conclude that AGXX^®^ is an effective antimicrobial substance which might also act as a biofilm inhibitor based on our molecular data. The mechanism of inhibition is likely *ica*-independent without the production of PIA, by interfering with the QS system and by repressing genes associated with surface adhesin and lipopolysaccharide synthesis. In addition, the antimicrobial might also reduce pathogenesis of *S. aureus* 04-02981 by down-regulating the synthesis of toxins and virulence factors.

## Author Contributions

AV performed all the microbiological and molecular experiments, drafted the manuscript, and designed the figures. AdJ supervised and discussed bioinformatics analyses of RNA-seq, and prepared and deposited the RNA-seq data at NCBI. DW performed the confocal microscopy and analyzed the data. JK drafted part of the discussion and gave insightful suggestions on molecular biology of Gram-positive pathogens. EG designed the project and supervised all the experiments. All authors discussed and corrected the manuscript.

## Conflict of Interest Statement

The authors declare that the research was conducted in the absence of any commercial or financial relationships that could be construed as a potential conflict of interest.

## Footnotes

1 http://www.systatsoftware.com

2 https://www.ncbi.nlm.nih.gov/geo/query/acc.cgi?acc=GSE103064

3 www.systatsoftware.com

## Supplementary Material

**FIGURE S1 |** Library sizes of all the RNA samples (*S. aureus* 04-02981, *S. aureus* 04-02981 + Ag, and *S. aureus* 04-02981 + AGXX), at different time periods. The image indicates the read depth of each sample. The *X*-axis represents the experiment names as used in the factors file, and gene counts file during RNA-seq analysis via T-REx. The sample names comprise the metal sheet used, followed by the time of exposure. For example, sample ‘AGXX_06’ represents *S. aureus* 04-02981 exposed to AGXX for 6 minutes. The *Y*-axis represents the total number of mapped reads.

**TABLE S1 |** Primer and probe sequences used for RT-qPCR.

**TABLE S2 |** AGXX^®^-mediated growth inhibition of *S. aureus* 04-02981 in batch cultures.

**TABLE S3 |** Alignment rates of the RNA-sequences of *S. aureus* 04-02981.

**TABLE S4 |** Differentially expressed genes in *S. aureus* 04-02981 on exposure to Ag, and AGXX^®^.

**TABLE S5 |** Gene Ontology assignments on exposing *S. aureus* 04-02981 to AGXX^®^ for 6 minutes. Rate = The rating values (1 to 5) reflect binned values based on: (TopHits/ClassSize) ^∗^ -log2(adj-pvalue).

**TABLE S6 |** Gene Ontology assignments on exposing *S. aureus* 04-02981 to Ag for 80 minutes. Rate = The rating values (1 to 5) reflect binned values based on: (TopHits/ClassSize) ^∗^ -log2(adj-pvalue).

**TABLE S7 |** Expression of operons in *S. aureus* 04-02981 on exposure to AGXX^®^ for 6 minutes. Rate = The rating values (1 to 5) reflect binned values based on: (TopHits/ClassSize) ^∗^ -log2(adj-pvalue).

**TABLE S8 |** Expression of operons in *S. aureus* 04-02981 on exposure to Ag for 80 minutes. Rate = The rating values (1 to 5) reflect binned values based on: (TopHits/ClassSize) ^∗^ -log2(adj-pvalue).

## References

[B1] Arciola C. R. Baldassarri L. Montanaro L. (2001). Presence of *icaA* and *icaD* genes and slime production in a collection of staphylococcal strains from catheter-associated infections. *J. Clin. Microbiol.* 39 2151–2156. 11376050 10.1128/JCM.39.6.2151-2156.2001 PMC88104

[B2] Arciola C. R. Campoccia D. Ravaioli S. Montanaro L. (2015). Polysaccharide intercellular adhesin in biofilm: structural and regulatory aspects. *Front. Cell. Infect. Microbiol.* 5:7. 10.3389/fcimb.2015.00007 25713785 PMC4322838

[B3] Baker J. Sitthisak S. Sengupta S. Johnson M. Jayaswal R. K. Morrissey J. A. (2010). Copper stress induces a global stress response in *Staphylococcus aureus* and represses *sae* and *agr* expression and biofilm formation. *Appl. Environ. Microbiol.* 76 150–160. 10.1128/AEM.02268-09 19880638 PMC2798663

[B4] Baldry M. Nielsen A. Bojer M. S. Zhao Y. Friberg C. Ifrah D. (2016). Norlichexanthone reduces virulence gene expression and biofilm formation in *Staphylococcus aureus*. *PLOS ONE* 11:e0168305. 10.1371/journal.pone.0168305 28005941 PMC5179057

[B5] Barbu E. M. Mackenzie C. Foster T. J. Höök M. (2014). SdrC induces staphylococcal biofilm formation through a hemophilic interaction. *Mol. Microbiol.* 94 172–185. 10.1111/mmi.12750 25115812 PMC5718044

[B6] Bouchard A. (2011). AgXX Glass Microspheres. In Vitro Evaluation of Cytotoxicity by Neutral Red Assay Using MRC-5 Cell Line with a Direct Contact Procedure. Report 20100326STP Dresden: APOGEPHA Arzneimittel GmbH.

[B7] Chen C. Krishnan V. Macon K. Manne K. Schneewind O. (2013). Secreted proteases control autolysin-mediated biofilm growth of *Staphylococcus aureus*. *J. Biol. Chem.* 288 29440–29452. 10.1074/jbc.M113.502039 23970550 PMC3795244

[B8] Cheung G. Y. C. Wang R. Khan B. A. Sturdevant D. E. Otto M. (2011). Role of the accessory gene regulator *agr* in community- associated methicillin- resistant *Staphylococcus aureus* pathogenesis. *Infect. Immun.* 79 1927–1935. 10.1128/IAI.00046-11 21402769 PMC3088142

[B9] Chopra I. (2007). The increasing use of silver-based products as antimicrobial agents: a useful development or a cause for concern? *J. Antimicrob. Chemother.* 59 587–590. 10.1093/jac/dkm006 17307768

[B10] Clauss-Lendzian E. Vaishampayan A. de Jong A. Landau U. Meyer C. Kok J. (2017). Stress response of a clinical *Enterococcus faecalis* isolate subjected to a novel antimicrobial surface coating. *Microbiol. Res.* 10.1016/j.micres.2017.11.006 29458868

[B11] de Jong A. van der Meulen S. Kuipers O. P. Kok J. (2015). T-REx: transcriptome analysis webserver for RNA-seq expression data. *BMC Genomics* 16:663. 10.1186/s12864-015-1834-4 26335208 PMC4558784

[B12] Ellis M. W. Schlett C. D. Millar E. V. Crawford K. B. Cui T. Lanier J. B. (2014). Prevalence of nasal colonization and strain concordance in patients with community-associated *Staphylococcus aureus* skin and soft- tissue infections. *Infect. Control Hosp. Epidemiol.* 35 1251–1256. 10.1086/678060 25203178 PMC5824647

[B13] Fitzpatrick F. Humphreys H. O’Gara J. P. (2005). Evidence for *icaADBC*-independent biofilm development mechanism in methicillin-resistant *Staphylococcus aureus* clinical isolates. *J. Clin. Microbiol.* 43 1973–1976. 15815035 10.1128/JCM.43.4.1973-1976.2005 PMC1081404

[B14] Frees D. Chastanet A. Qazi S. Sorensen K. Hill P. Msadek T. (2005). Clp ATPases are required for stress tolerance, intracellular replication and biofilm formation in *Staphylococcus aureus*. *Mol. Microbiol.* 54 1445–1462. 10.1111/j.1365-2958.2004.04368.x 15554981

[B15] Gouaux A. Hobaugh M. Song L. (1997). α-hemolysin, γ-hemolysin, and leukocidin from *Staphylococcus aureus*: distant in sequence but similar in structure. *Protein Sci.* 6 2631–2635. 10.1002/pro.5560061216 9416613 PMC2143621

[B16] Grass G. Rensing C. Solioz M. (2011). Metallic copper as an antimicrobial surface. *Appl. Environ. Microbiol.* 77 1541–1547. 10.1128/AEM.02766-10 21193661 PMC3067274

[B17] Gupta A. Matsui K. Lo J.-F. Silver S. (1999). Molecular basis for resistance to silver cations in *Salmonella*. *Nat. Med.* 5 183–188. 10.1038/5545 9930866

[B18] Guridi A. Diederich A. K. Aguila-Arcos S. Garcia-Moreno M. Blasi R. Broszat M. (2015). New antimicrobial contact catalyst killing antibiotic resistant clinical and water borne pathogens. *Mater. Sci. Eng. C Mater. Biol. Appl.* 50 1–11. 10.1016/j.msec.2015.01.080 25746238

[B19] Hammer N. D. Skaar E. P. (2011). Molecular mechanisms of *Staphylococcus aureus* iron acquisition. *Annu. Rev. Microbiol.* 65 129–147. 10.1146/annurev-micro-090110-102851 21639791 PMC3807827

[B20] Huebner J. Wang Y. Krueger W. A. Madoff L. C. Martirosian G. Boisot S. (1999). Isolation and chemical characterization of a capsular polysaccharide antigen shared by clinical isolates of *Enterococcus faecalis* and vancomycin-resistant *Enterococcus faecium*. *Infect. Immun.* 67 1213–1219. 10024563 10.1128/iai.67.3.1213-1219.1999 PMC96449

[B21] Ibarra J. A. Pérez-Rueda E. Carroll R. K. Shaw L. N. (2013). Global analysis of transcriptional regulators in *Staphylococcus aureus*. *BMC Genomics* 14:126. 10.1186/1471-2164-14-126 23442205 PMC3616918

[B22] Karatsa-Dodgson M. Woermann M. E. Gruendling A. (2010). *In vitro* analysis of the *Staphylococcus aureus* lipoteichoic acid synthase enzyme using fluorescently labeled lipids. *J. Bacteriol.* 192 5341–5349. 10.1128/JB.00453-10 20709894 PMC2950504

[B23] Kirmusaoglu S. (2016). “Staphylococcal biofilms: pathogenicity, mechanism and regulation of biofilm formation by Quorum-Sensing system and antibiotic resistance mechanisms of biofilm-embedded microorganisms,” in *Microbial Biofilms - Importance and Applications*, ed. Dhanasekaran D. (Rijeka: In Tech).

[B24] Kluytmans J. van Belkum A. Verbrugh H. (1997). Nasal carriage of *Staphylococcus aureus*: epidemiology, underlying mechanisms, and associated risks. *Clin. Microbiol. Rev.* 10 505–520. 9227864 10.1128/cmr.10.3.505 PMC172932

[B25] Landau U. (2013). AGXX - Eine nachhaltige lösung für die Entkeimung wässriger Lösungen. *Galvanotechnik* 11 2169–2184.

[B26] Landau U. Meyer C. Grohmann E. (2017a). AGXX – Beitrag der Oberflächentechnik zur Vermeidung von Biofilmen (Teil 1). *Galvanotechnik* 108 885–890.

[B27] Landau U. Meyer C. Grohmann E. (2017b). AGXX – Beitrag der Oberflächentechnik zur Vermeidung von Biofilmen (Teil 2). *Galvanotechnik* 108 1110–1121.

[B28] Landsdown A. B. G. (2010). A pharmacological and toxicological profile of silver as an antimicrobial agent in medical devices. *Adv. Pharmacol. Sci.* 2010:910686. 10.1155/2010/910686 21188244 PMC3003978

[B29] Langmead B. Salzberg S. (2012). Fast gapped-read alignment with Bowtie 2. *Nat. Methods* 9 357–359. 10.1038/nmeth.1923 22388286 PMC3322381

[B30] Li H. Handsaker B. Wysoker A. Fennell T. Ruan J. Homer N. (2009). The sequence alignment/map (SAM) format and SAMtools. *Bioinformatics* 25 2078–2079. 10.1093/bioinformatics/btp352 19505943 PMC2723002

[B31] Lister J. L. Horswill A. R. (2014). *Staphylococcus aureus* biofilms: recent developments in biofilm dispersal. *Front. Cell. Infect. Microbiol.* 4:178. 10.3389/fcimb.2014.00178 25566513 PMC4275032

[B32] Liu Q. Yo W.-S. Bae T. (2016). The SaeRS two-component system of *Staphylococcus aureus*. *Genes* 7:81. 10.3390/genes7100081 27706107 PMC5083920

[B33] Loo C.-Y. Rohanizadeh R. Young P. M. Traini D. Cavaliere R. Whitchurch C. B. (2016). Combination of silver nanoparticles and curcumin nanoparticles for enhanced anti-biofilm activities. *J. Agric. Food Chem.* 64 2513–2522. 10.1021/acs.jafc.5b04559 26595817

[B34] Ma Y. Xu Y. Yestrepsky B. D. Sorenson R. J. Chen M. Larsen S. D. (2012). Novel inhibitors of *Staphylococcus aureus* virulence gene expression and biofilm formation. *PLOS ONE* 7:e47255. 10.1371/journal.pone.0047255 23077578 PMC3471953

[B35] Maillard J. Hartemann P. (2013). Silver as an antimicrobial: facts and gap in knowledge. *Crit. Rev. Microbiol.* 39 373–383. 10.3109/1040841X.2012.713323 22928774

[B36] Marathe N. P. Nagarkar S. S. Vaishampayan A. A. Rasane M. H. Samant S. A. Dohe V. (2015). High prevalence of class 1 integrons in clinical isolates of methicillin-resistant *Staphylococcus aureus* from India. *Indian J. Med. Microbiol.* 33 231–236. 10.4103/0255-0857.154905 25865973

[B37] Missineo A. Poto D. A. Geoghegan J. A. Rindi S. Heilbronner S. Gianotti V. (2014). IsdC from *Staphylococcus lugdunensis* induces biofilm formation under low-iron growth conditions. *Infect. Immun.* 82 2448–2459. 10.1128/IAI.01542-14 24686057 PMC4019187

[B38] Naas T. Coignard B. Carbonne A. Blanckaert K. Bajolet O. Bernet C. (2006). VEB-1 Extended-spectrum beta-lactamase-producing *Acinetobacter baumannii*, France. *Emerg. Infect. Dis.* 12 1214–1222. 10.3201/eid1208.051547 16965700 PMC3291215

[B39] Njoroge J. Sperandio V. (2009). Jamming bacterial communication: new approaches for the treatment of infectious diseases. *EMBO Mol. Med.* 1 201–210. 10.1002/emmm.200900032 20049722 PMC2801573

[B40] Nuebel U. Dordel J. Kurt K. Strommenger B. Westh H. Shukla S. K. (2010). A timescale for evolution, population expansion, and spatial spread of an emerging clone of methicillin resistant *Staphylococcus aureus*. *PLOS Pathog.* 6:e1000855. 10.1371/journal.ppat.1000855 20386717 PMC2851736

[B41] Nyenje M. E. Green E. Ndip R. N. (2013). Evaluation of the effect of different growth media and temperature on the suitability of biofilm formation by *Enterobacter cloacae* strains isolated from food samples in South Africa. *Molecules* 18 9582–9593. 10.3390/molecules18089582 23966079 PMC6270404

[B42] Paniagua-Contreras G. Sáinz- Espuñes T. Monroy-Pérez E. Rodríguez-Moctezuma J. R. Arenas-Aranda D. Negrete-Abascal E. (2012). Virulence markers in *Staphylococcus aureus* strains isolated from hemodialysis catheters of Mexican patients. *Adv. Microbiol.* 2 476–487. 10.4236/aim.2012.24061

[B43] Paraje M. G. (2011). “Antimicrobial resistance in biofilms,” in *Science against Microbial Pathogens: Communicating Current Research and Technological Advances* Vol. 2 ed. Mendez-Vilas A. (Badajoz: Formatex Research Center), 736–744.

[B44] Probst I. Vaishampayan A. Kuechler V. Grohmann E. (2016). Antimikrobielle Oberflächenbeschichtung tötet multiresistente Krankheitserreger. *Flug Reisemed.* 23 14–17.

[B45] Qin N. Tan X. Jiao Y. Liu L. Zhao W. Yang S. (2014). RNA-Seq-based transcriptome analysis of methicillin-resistant *Staphylococcus aureus* biofilm inhibition by ursolic acid and resveratrol. *Sci. Rep.* 4:5467. 10.1038/srep05467 24970710 PMC4073122

[B46] Quave C. L. Horswill A. R. (2014). Flipping the switch: tools for detecting small molecule inhibitors of staphylococcal virulence. *Front. Microbiol.* 5:706. 10.3389/fmicb.2014.00706 25566220 PMC4264471

[B47] Resch A. Rosenstein R. Nerz C. Goetz F. (2005). Differential gene expression profiling of *Staphylococcus aureus* cultivated under biofilm and planktonic conditions. *Appl. Environ. Microbiol.* 71 2663–2676. 15870358 10.1128/AEM.71.5.2663-2676.2005 PMC1087559

[B48] Sause W. E. Buckley P. T. Strohl W. R. Lynch A. S. Torres V. J. (2015). Antibody-based biologics and their promise to combat *Staphylococcus aureus* infections. *Trends Pharmacol. Sci.* 37 231–241. 10.1016/j.tips.2015.11.008 26719219 PMC4764385

[B49] Schäberle T. F. Hack I. M. (2014). Overcoming the current deadlock in antibiotic resistance. *Trends Microbiol.* 22 165–167. 10.1016/j.tim.2013.12.007 24698433

[B50] Schiwon K. Arends K. Rogowski K. M. Fuerch S. Prescha K. Sakinc T. (2013). Comparison of antibiotic resistance, biofilm formation and conjugative transfer of *Staphylococcus* and *Enterococcus* isolates from International Space Station and Antarctic research station Concordia. *Microb. Ecol.* 65 638–651. 10.1007/s00248-013-0193-4 23411852

[B51] Smith K. Gould K. A. Gordon R. Gemmell C. G. Hinds J. Lang S. (2010). Influence of tigecycline on expression of virulence factors in biofilm- associated cells of methicillin- resistant *Staphylococcus aureus*. *Antimicrob. Agents Chemother.* 54 380–387. 10.1128/AAC.00155-09 19858261 PMC2798542

[B52] Sully E. K. Malachowa N. Elmore B. O. Alexander S. M. Femling J. K. Gray B. M. (2014). Selective chemical inhibition of *agr* Quorum Sensing in *Staphylococcus aureus* promotes host defense with minimal impact on resistance. *PLOS Pathog.* 10:e1004174. 10.1371/journal.ppat.1004174 24945495 PMC4055767

[B53] Trapnell C. Williams B. A. Pertea G. Mortazavi A. Kwan G. van Baren M. J. (2010). Transcript assembly and quantification by RNA-Seq reveals unannotated transcripts and isoform switching during cell differentiation. *Nat. Biotechnol.* 28 511–515. 10.1038/nbt.1621 20436464 PMC3146043

[B54] Tuchscherr L. Loeffler B. Buzzola F. R. Sordelli D. O. (2010). *Staphylococcus aureus* adaptation to the host and persistence: role of loss of capsular polysaccharide expression. *Future Microbiol.* 5 1823–1832. 10.2217/fmb.10.147 21155664

[B55] Warnes S. L. Keevil C. W. (2013). Inactivation of norovirus on dry copper alloy surfaces. *PLOS ONE* 8:e75017. 10.1371/journal.pone.0075017 24040380 PMC3767632

[B56] Wass Y. A. (2009). SigmaPlot 11: Now with total sigmaStat integration. *Sci. Comput.* 26 21.

[B57] Wu Y. Wu Y. Zhu T. Han H. Liu H. Xu T. (2015). *Staphylococcus epidermidis* SrrAB regulates bacterial growth and biofilm formation differently under oxic and microaerobic conditions. *J. Bacteriol.* 197 459–476. 10.1128/JB.02231-14 25404696 PMC4285975

[B58] Xu L. Li H. Vuong C. Vadyvaloo V. Wang J. Yao Y. (2006). Role of the *luxS* Quorum-Sensing system in biofilm formation and virulence of *Staphylococcus epidermidis*. *Infect. Immun.* 74 488–496. 16369005 10.1128/IAI.74.1.488-496.2006 PMC1346618

[B59] Yarwood J. M. Bartels D. J. Volper E. M. Greenberg E. P. (2004). Quorum Sensing in *Staphylococcus aureus* biofilms. *J. Bacteriol.* 186 1838–1850. 10.1128/JB.186.6.1838-1850.2004 14996815 PMC355980

