# Supplementary figures and images for: A Novel Antimicrobial Coating Represses Biofilm and Virulence-Related Genes in Methicillin-Resistant Staphylococcus aureus
Source: Front Microbiol. 2018 Feb 15;9:221. doi: 10.3389/fmicb.2018.00221 (PMC5818464; doi:10.3389/fmicb.2018.00221)

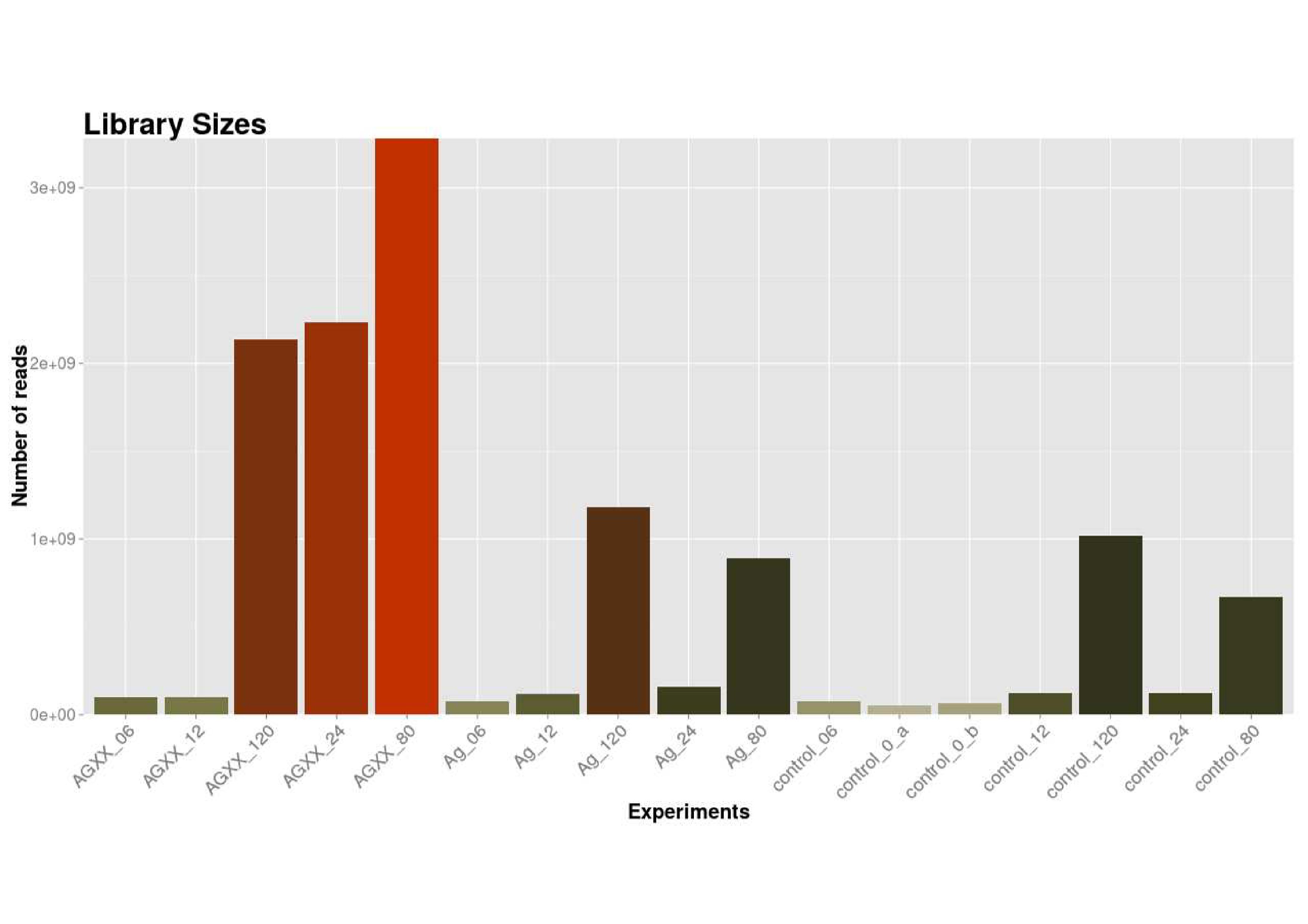

Supplement: Supplementary file 1 [file Image_1.JPEG]
